# Rheology, Printability, and Texture of Extrusion-Based 3D-Printed Self-Supporting Soft Gels Formulated with Pea and Chickpea Proteins

**DOI:** 10.3390/foods15132394

**Published:** 2026-07-06

**Authors:** Marco Menegon, Laura Piazza

**Affiliations:** Department of Environmental Science and Policy, Università degli Studi di Milano, Via Mangiagalli 25, 20133 Milan, Italy; marco.menegon@unimi.it

**Keywords:** 3D food printing, rheology, printability

## Abstract

Predicting printability and final texture in extrusion-based 3D printing of soft, self-supporting food gels remains challenging, particularly when realistic plant-based ingredients are used instead of simplified model systems. In this study, two plant protein–hydrocolloid inks based on commercial pea protein isolate (PPI) and chickpea protein concentrate (CPC) were developed and compared within the same hydrocolloid framework (0.36% low-acyl gellan gum and 1.00% xanthan gum). The formulations differed in protein ingredient level, moisture content, sorbitol concentration, and salt origin, allowing evaluation of how complete formulation design governs rheology, printability, and texture. The CPC-based ink showed higher yield stress than the PPI-based ink (158.10 ± 18.17 vs. 119.56 ± 18.84 Pa), whereas both inks exhibited similar shear-thinning behavior (*n* ≈ 0.35). Thixotropic recovery at 60 °C was limited in both systems (16–19%), while oscillatory tests revealed weak-gel behavior, with higher Bohlin gel strength for the PPI-based ink (65.69 ± 5.59 vs. 38.97 ± 2.08 kPa). Both formulations enabled continuous extrusion and the fabrication of self-supporting printed objects, although geometric fidelity of the internal infill remained limited, particularly in CPC samples. Compression testing showed that CPC gels were slightly stiffer and tougher, whereas PPI gels were more resistant to irreversible deformation. Overall, the results indicate that when commercial ingredients are used for food 3D printing purposes, rheology, printability, and final texture were governed primarily by the formulation design, rather than by protein source alone.

## 1. Introduction

Three-dimensional (3D) food printing has emerged as a promising strategy for producing texture-modified foods tailored to individuals with specific oral processing needs, where precise control of flow behavior, structural integrity, and deformation under low stress conditions is essential [1,2,3]. For personalized diets aimed at vulnerable populations, including individuals with chewing or swallowing impairments, 3D printing enables the production of self-supporting soft solids as an alternative to conventional purees or molded products, which often rely on high starch content and provide a relatively uniform, gelatinous mouthfeel which may lack sensory diversity in appearance and mouthfeel. By leveraging tailored biopolymer and hydrocolloid systems, printed foods can achieve targeted textures while supporting improved sensory design [4].

In extrusion-based 3D printing systems, the performance of the printed construct is governed by the rheological and physicochemical properties of the food ink, together with process parameters. Therefore, rational formulation of these composite biomaterials is essential to ensure both printability and compliance with texture-modified dietary requirements. A balance between extrusion flowability, shape fidelity, and soft-gel deformability must be achieved [5].

From a structuring perspective, suitable food inks must flow through the nozzle under shear and rapidly recover their structure after deposition [6]. Hydrocolloids play a key role in structuring printable soft gels. Among gelling agents, low-acyl gellan promotes ion-mediated double-helix aggregation and the formation of well-defined junction zones, resulting in elastic and mechanically resistant networks. In contrast, high-acyl gellan increases flexibility and reduces brittleness due to acyl substituents that hinder tight helix aggregation [7,8,9]. Starch-based inks also exhibit shear-thinning behavior, facilitating extrusion while maintaining structural integrity after deposition [10]. Among thickeners, carboxymethylcellulose (CMC), an anionic water-soluble cellulose derivative, contributes to viscosity enhancement, shear-thinning behavior, and water retention, supporting extrusion stability and interlayer adhesion [11].

From a nutritional perspective, potential users of 3D-printed soft foods often experience protein deficiencies or muscle impairment. Protein density is therefore a key consideration. Owing to the increasing demand for plant proteins, plant-based products have attracted considerable attention from consumers. As highlighted by McClements in previous work [12], 3D food printing is particularly relevant in the plant-based sector, which offers sustainable protein sources. Plant proteins such as pea (*Pisum sativum*) and chickpea (*Cicer arietinum*) provide functional structuring capabilities, including water binding and network formation through thermal denaturation and protein–polysaccharide interactions. Pea and chickpea proteins are highly expected to interact with low-acyl (LA) gellan and xanthan, typically forming a complexed, highly structured, and synergistic network [13,14]. Their globular structure allows unfolding and intermolecular association, enhancing gel strength and bolus cohesiveness. Additionally, electrostatic interactions between proteins and negatively charged polysaccharides can modulate viscoelastic behavior, improving structural stability without excessive hardness [15,16]. The combination of legume proteins and hydrocolloids thus enables the design of composite inks with tunable rheological and mechanical properties in addition to a suitable nutritional profile.

A major challenge for extrusion-based 3D printing remains the prediction and control of printability and shape fidelity. These are governed by the interplay between ink rheology and process conditions. Key rheological parameters—including viscosity, yield stress, and viscoelastic moduli are critical for extrusion behavior, structural recovery, and dimensional stability [17,18,19,20]. Successful inks typically exhibit shear-thinning behavior and yield stress, behaving as soft solids at rest while flowing under stress. Higher storage modulus is generally associated with improved shape fidelity, whereas suboptimal conditions can lead to deformation and reduced accuracy [21]. Recent studies have further highlighted the importance of elastic-dominant behavior, shear-thinning properties, and post-printing treatments in determining final structure [22,23,24]. Despite these advances, robust and transferable criteria linking formulation and process variables to printing outcomes remain limited, particularly for soft, viscoelastic, self-supporting systems.

Within this context, the present study investigates extrusion-based, layer-by-layer fabrication of self-supporting soft gels using commercially relevant plant protein ingredients and a common hydrocolloid backbone. Rather than comparing purified protein systems under composition-normalized conditions, the study evaluates two complete formulations based on commercial pea protein isolate and chickpea protein concentrate, structured with low-acyl gellan gum and xanthan gum. This design reflects practical formulation constraints and industrially relevant ingredient use, while enabling analysis of how formulation architecture affects rheology, printability, and final mechanical response.

The novelty of the work lies in combining realistic plant-based formulations with a multilevel characterization strategy linking steady-shear flow, thixotropic recovery, weak-gel oscillatory behavior, printing performance, and compression texture of stabilized printed objects. The aim of the study was therefore not to isolate protein source as a single independent variable, but to determine how complete formulation design governs extrusion behavior, post-deposition stabilization, and the texture of self-supporting 3D-printed soft gels.

## 2. Materials and Methods

### 2.1. Food Inks Preparation

Food inks were formulated using commercially available plant-based protein ingredients as the primary structuring components. It should be noted that these were not purified proteins, but commercial pea protein isolate and chickpea protein concentrate, which differ in protein content and compositional profile. Consequently, the amount of each ingredient incorporated in the formulations was adjusted to achieve comparable protein levels, resulting in different mass fractions of the two ingredients in the final inks.

Xanthan gum and low-acyl gellan gum were used as structuring agents, and sorbitol was included as a plasticizer. NaCl was present at comparable levels in both formulations, although its origin differed, as described below.

Preliminary experimental trials, not reported here in detail, were carried out to identify formulations satisfying three practical requirements simultaneously: (i) comparable effective protein contribution in the final inks, (ii) continuous extrusion through the selected nozzle without clogging or filament interruption, and (iii) sufficient post-deposition self-support to obtain multilayer printed objects. The formulation work was organized in three main stages: characterization of the protein ingredients, development of the ink preparation protocol, and optimization of the thermal and printing setup. Within this framework, ingredient amounts were initially selected to approach 10 g of effective protein in the ink, based on Kjeldahl measurements, and were subsequently adjusted through preliminary trials until printable systems were obtained. Table 1 summarizes the main outcomes obtained.

The following two bioink formulations were set: the first ink was based on commercial pea protein isolate (12.50% *w*/*w*), combined with low-acyl gellan gum (0.36% *w*/*w*), xanthan gum (1.00% *w*/*w*), and sorbitol (2.20% *w*/*w*). The second ink was formulated using chickpea protein concentrate (14.71% *w*/*w*), with the same concentrations of low-acyl gellan gum (0.36% *w*/*w*), xanthan gum (1.00% *w*/*w*), and lower sorbitol content (0.36% *w*/*w*).

The target was to obtain comparable protein levels in the final formulations using commercially available protein ingredients with different protein purity. For this reason, different ingredient mass fractions were required for PPI and CPC, resulting in differences not only in protein ingredient load but also in water content, sorbitol level, and salt origin.

In both formulations, the NaCl concentration was comparable (0.225% *w*/*w*). Nevertheless, in the pea protein-based ink, sodium chloride was added during preparation, whereas in the chickpea-based ink, the salt was already present in the protein concentrate and was therefore not added separately.

Pea protein isolate and chickpea protein concentrate were supplied by Deimos Srl (Milan, Italy), while xanthan gum and low-acyl gellan gum were provided by CP Kelco (Atlanta, GA, USA). Sorbitol was purchased from Sigma-Aldrich (St. Louis, MO, USA).

Food ink preparation followed a protocol refined through preliminary optimization trials. Protein ingredients and hydrocolloids were first dispersed in water at 60 °C using an Ultra-Turrax mixer (T25, 18G disperser, IKA, Staufen im Breisgau, Germany) for 3 min at 200 rpm. The dispersion was then subjected to sonication (DU-100 Digital Ultrasonic Cleaner, ARGO Lab, Carpi, MO, Italy) at 60 °C for 5 min. Subsequently, the gelling agent and sorbitol were dissolved using a magnetic stirrer (MS-H340-S4, DLAB Scientific Co., Beijing, China) and combined with the protein dispersion at 62 °C.

The pH of the inks was measured in triplicate using a pHenomenal pH 1100L pH meter (VWR International, Radnor, PA, USA). The pea protein-based ink showed a pH of 7.2., whereas the chickpea protein-based ink showed a pH of 7.5.

### 2.2. 3D Printing Procedure

A pressure-controlled, syringe-based extrusion 3D printer (Procusini^®^ Research 3D Food Printer, Freising, Germany) was used to fabricate computer-aided designed structures through a layer-by-layer deposition approach. The printer was equipped with a heating system to maintain the temperature of the ink in both the syringe and the nozzle.

A nozzle with a diameter of 1.5 mm was used, and the layer height was set at 1.2 mm. The syringe temperature was maintained at 60 °C. Before printing, the loaded syringe was pre-heated for 20 min at 60 °C in order to minimize thermal fluctuations during loading and to maintain the ink in a sufficiently fluid state prior to extrusion. This step was necessary because preliminary trials showed that even moderate cooling during loading could trigger premature gelation inside the cartridge, leading to nozzle clogging and loss of printability. The print speed was set 8 mm/s, while the extrusion flow rate was fixed at 80%. The printed geometry was designed using Fusion 360 (v2.0.20981, Autodesk, 2024) and consisted of a square structure (20 × 20 × 10 mm) with a grid infill density of 20%. The bed temperature was not actively controlled and remained at ambient temperature.

These conditions ensured that the food ink remained sufficiently fluid during extrusion while allowing rapid gelation immediately after deposition, as the system operated close to the sol–gel transition range of low-acyl gellan. Preliminary tests also showed that printing below this thermal condition was not feasible because of gelation within the cartridge and consequent nozzle clogging. The selected infill density was chosen as a compromise between structural stability and the need to maintain an internal architecture sensitive enough to reveal differences in ink printability.

### 2.3. Techno-Functional Properties of Protein Ingredients

Techno-functional properties of protein-rich commercial flours, including water holding capacity (WHC) (Equation (1)), oil holding capacity (OHC) (Equation (2)) [25], foaming capacity (FC) (Equation (3)), emulsion capacity (EC) (Equation (4)), and emulsion stability (ES) (Equation (5)) [26] were evaluated as follows:

(1) $$WHC\left(\%\right)=\frac{WHS-WDS}{WDS}\times 100,$$

where WHS means weight of hydrated sample and WDS represents weight of dry sample.

(2) $$OHC\left(\%\right)=\frac{WSO-WS}{WS}\times 100,$$

where WSO means weight of sample with oil and WS represent weight sample without oil.

(3) $$FC\left(\%\right)=\frac{VAM-IV}{IV}\times 100,$$

where VAM means volume after homogenization and IV represents initial volume.

(4) $$EC\left(\%\right)=\frac{HEL}{TVH}\times 100,$$

where HEL means height of emulsified layer and TVH represents total volume height.

(5) $$ES\left(\%\right)=\frac{HELAH}{TVH}\times 100,$$

where HELAH means height of emulsified layer after heating and TVH represents total volume height.

Protein ingredients’ total solids (TS) content was determined using a thermogravimetric moisture analyzer (Kern DAB 100-3, Balingen, Germany) at 105 °C until constant weight was achieved. Powdered ingredients’ protein content was quantified by the Kjeldahl method according to AOAC Official Method 984.13 [27]. A nitrogen-to-protein conversion factor of 6.25 was applied as a conventional factor for legume-based ingredients, and the results are reported here as comparative values for formulation purposes.

The moisture content of both food inks and printed objects was measured using a thermogravimetric moisture analyzer (Kern DAB 100-3, Balingen, Germany) at 105 °C until constant weight was achieved. Food inks were analyzed immediately after preparation at 60 °C. Printed objects were analyzed after overnight storage at 4 °C, followed by equilibration to room temperature.

### 2.4. Flow Behavior of Inks

The flow behavior of inks was characterized by shear flow tests performed with a CMT rheometer DHR-2 (TA Instruments, New Castle, DE, USA) equipped with a 40 mm cone-plate geometry, at a 28 µm gap. Samples were subjected to shear rates ranging from 0.1 to 100 s^−1^, at 60 °C. Flow behavior was modeled using the Herschel–Bulkley model (Equation (6)), which describes the pseudoplastic behavior of yield-stress fluids [28].

(6) $$\sigma =\sigma_{0}+k{\dot{\gamma }}^{n},$$

where σ is the shear stress (Pa), σ_0_ is the yield stress (Pa), k is the consistency index (Pa·s^n^), $\dot{\gamma }$ is the shear rate (s^−1^) and n is the flow behavior index (dimensionless).

The flow behavior was analyzed through the software TRIOS 5.8 (TA Instruments, USA). Analyses were conducted on aliquots collected from three independent pea protein-based inks and three independent chickpea protein-based inks at 60 °C, immediately after preparation and before the printing stage.

### 2.5. Viscoelastic Behavior of Inks

Viscoelastic properties of the inks were evaluated at 60 °C using a CMT rheometer DHR-2 (TA Instruments, New Castle, DE, USA) equipped with a 40 mm plate geometry, at a 100 µm gap. A strain sweep test (0.01–100% strain at 1 Hz) was first performed to identify the linear viscoelastic region (LVR). Subsequently, a frequency sweep test from 0.1 to 100 Hz was carried out within the LVR (strain = 0.5%). The obtained mechanical spectra (G′ (Pa) and G″ (Pa) versus frequency (Hz) were analyzed using TRIOS 5.8 software. The Bohlin power law model (Equation (7)), that is validated for weak gels [29], was used to describe the frequency-dependent viscoelastic behavior under oscillatory shear.

(7) G*=G′2+G″2=Afω1z,

where G^*^ is the complex modulus (Pa), G′ is the storage modulus (Pa), G″ is the loss modulus (Pa), A_f_ is gel strength, ω is the angular frequency and Z represents degree of interaction.

Analyses were conducted on aliquots collected from three independent pea protein–based inks and three independent chickpea protein-based inks at 60 °C, immediately after preparation and before the printing stage.

### 2.6. Thixotropic Behavior of Inks

Thixotropic behavior of the food inks was investigated using a CMT rheometer DHR-2 (TA Instruments, New Castle, DE, USA) equipped with a 40 mm cone-plate geometry, at a 28 µm gap. A solvent trap was used to prevent loss of sample. The viscosity recovery of the samples was recorded using the 3 Intervals Thixotropy Test (3ITT), applying the following shear rate regimes: 0.1 s^−1^ in the first and third intervals (each lasting 60 s), and 100 s^−1^ in the second interval (lasting 120 s) [30]. The test consisted of three sequential intervals designed to assess structural breakdown and recovery under shear. The recovery ratio was calculated as the percentage ratio between the viscosity measured at the end of the third interval and the viscosity measured at the end of the first interval, before the application of high shear (Equation (8)).

(8) $$Recovery\;Ratio\;(\%)=\frac{\eta_{f}}{\eta_{0}}\times 100,$$

where η_f_ is the viscosity (Pa·s) measured during the third interval and η_0_ is the initial viscosity (Pa·s).

The analysis was performed at 60 °C immediately after preparation, prior to the printing stage.

### 2.7. Instrumental Texture of 3D Printed Objects

Mechanical properties of the printed samples were assessed after overnight stabilization at 4 °C in a sealed jar, using a TA-XT2 texture analyzer (Stable Microsystems Ltd., Godalming, UK) equipped with a 75 mm diameter compression plate geometry. Tests were conducted at room temperature (21 °C). A uniaxial compression test was carried out until 70% deformation at test speed equal to 1 mm/s [31]. From the force/distance curve, the following parameters were evaluated: elastic modulus (Pa), yield stress (kPa), specific deformation energy (kJ/m^3^). The reported mechanical results were obtained from 30 measurements per formulation, corresponding to six printed samples collected from each of five independent preparation batches (AR1–AR5), for each of the two inks.

### 2.8. Fork Test

Printed objects were qualitatively assessed using the IDDSI fork pressure test as a descriptive reference for texture [32]. The observed deformation behavior was consistent with soft, deformable structures within the IDDSI Level 4–5 range.

### 2.9. Data Analysis

Statistical analysis was performed using JMP Pro version 18.0 (SAS Institute Inc., Cary, NC, USA). The mechanical properties obtained from the compression curves were evaluated for five cartridges (AR1–AR5) in both pea and chickpea-based formulations. For each cartridge, six independent samples were analyzed and used as experimental replicates. The raw data from individual replicates were used directly for statistical analysis. One-way analysis of variance (ANOVA) was applied, considering the cartridge (AR1–AR5) as the factor for comparison, followed by Tukey’s honestly significant difference (HSD) post hoc test (*p* < 0.05). Statistical analysis was performed separately for the pea-based formulations (AR1–AR5) and chickpea-based formulations (AR1–AR5), and subsequently on the combined dataset including both formulations, in order to (i) evaluate differences among cartridges within each formulation and (ii) compare the two formulations in terms of mechanical properties.

## 3. Results and Discussion

### 3.1. Ink Formulation

In the present study, the two ink systems differ not only in protein source but also in overall composition, including protein ingredient level, moisture content, and the origin of NaCl. These differences arise from the formulation strategy adopted, which aimed to achieve comparable nutritional targets and printing performance using commercially available, minimally refined ingredients rather than purified components. Accordingly, the present work does not aim to isolate the effect of protein sources as an independent variable. Instead, it compares two complete formulations, representative of realistic food systems, in which multiple compositional factors jointly contribute to the observed rheological, printing and mechanical behavior.

Table 2 reports the techno-functional properties of the two protein ingredients. PPI exhibited a markedly higher protein content than CPC (78.75 ± 0.35% vs. 68.50 ± 0.70%), while total solids were very similar (93.32 ± 0.33% for PPI and 94.14 ± 0.26% for CPC). As a direct consequence, different ingredient mass fractions were required to formulate the two inks at comparable protein targets. This resulted in different water contents in the final formulations, with moisture values of 69.44 ± 3.50 g/100 g for the PPI-based ink and 63.20 ± 3.93 g/100 g for the CPC-based ink. Thus, already at the formulation stage, the two inks differed not only in protein ingredient identity but also in solids concentration and hydration state, both of which are expected to influence flow and structural rebuilding [33].

The hydration-related properties reported in Table 2 further support this distinction. PPI showed a higher water-holding capacity than CPC (1.76 vs. 1.17 g H_2_O/g TS), indicating a greater ability to immobilize water within the matrix. From a printing perspective, this is relevant because more efficient water binding can facilitate the formation of hydrated and continuous structures, reduce water release during resting and deposition, and contribute to smoother filament formation. In contrast, the lower water-holding capacity of CPC suggests a less hydrated dispersed phase, which may contribute to a denser and more concentrated matrix under the same processing conditions. These trends are consistent with the known techno-functional behavior of legume proteins, whose hydration properties depend not only on botanical origin but also on ingredient purity, extraction history, and the relative contribution of major protein fractions. Pea proteins generally show strong water-binding capacity and good gel-forming ability, whereas chickpea proteins can display higher lipid interaction and formulation-dependent structuring behavior, especially after extraction-induced unfolding or partial denaturation [34,35,36,37].

The two ingredients also differed in interfacial functionality. PPI showed higher emulsifying capacity and emulsion stability than CPC, suggesting a greater ability to stabilize dispersed interfaces. Although the inks studied here were not emulsion-based systems, these parameters still indicate differences in surface activity and dispersion behavior, which may affect mixing homogeneity and polymer distribution in complex aqueous formulations. PPI also exhibited substantially higher foaming capacity than CPC, indicating a greater tendency to entrap air during mixing. This behavior represented a processing disadvantage, since excessive air incorporation can reduce structural homogeneity and weaken the printed object. For this reason, sonication was included in the preparation protocol to minimize entrapped air and improve dispersion uniformity.

Besides the protein ingredients, both inks were structured using the same concentrations of low-acyl gellan gum (0.36% *w*/*w*) and xanthan gum (1.00% *w*/*w*), while sorbitol was incorporated as a humectant and plasticizer. Low-acyl gellan gum was selected as the primary gelling agent because of its ability to form thermally stable, ion-mediated networks with sufficient rigidity to support layered architectures [7]. Xanthan gum was included to provide viscosity enhancement and shear-thinning behavior, thereby improving extrusion stability and multilayer cohesion [11,38]. Sorbitol contributed to moisture retention and matrix plasticization, but its concentration differed substantially between the two formulations: it was higher in the PPI-based ink (2.20% *w*/*w*) than in the CPC-based ink (0.36% *w*/*w*) and therefore should also be considered part of the formulation-specific architecture rather than a secondary additive. In xanthan-containing biopolymer systems, sorbitol has been shown to alter intermolecular interactions and increase matrix flexibility, supporting its interpretation here as a potentially relevant contributor to formulation-dependent rheological behavior [39].

Preliminary experimental trials, not reported here, enabled the identification of two formulations specifically suited to the printing capabilities of the Procusini 3D printer used in this study. The PPI-based ink contained 12.50% *w*/*w* pea protein isolate, 0.36% *w*/*w* low-acyl gellan gum, 1.00% *w*/*w* xanthan gum, and 2.20% *w*/*w* sorbitol. The CPC-based ink contained 14.71% *w*/*w* chickpea protein concentrate, 0.36% *w*/*w* low-acyl gellan gum, 1.00% *w*/*w* xanthan gum, and 0.36% *w*/*w* sorbitol. In both formulations, the overall NaCl concentration was comparable (0.225% *w*/*w*), but its origin differed: in the PPI-based ink, sodium chloride was added during preparation, whereas in the CPC-based system, the salt was already present in the protein ingredient and was therefore not added separately. This distinction may be relevant because salt not only contributes to ionic strength but may also influence local ion distribution during gellan structuring and, consequently, the balance between rigidity and flowability of the final ink.

Overall, the PPI-based ink combined a more hydrated matrix, higher foaming tendency, and higher sorbitol content, whereas the CPC-based ink relied on a higher protein ingredient load and lower moisture content within the same hydrocolloid framework. These differences define two distinct formulation architectures, and the rheological behavior discussed in the following section should therefore be interpreted as the outcome of complete ink design rather than as the isolated effect of protein source alone.

### 3.2. Rheology of Inks

Consistent with the formulation rationale outlined in Section 3.1, the rheological differences observed between the two inks should be interpreted as matrix-level responses arising from differences in protein ingredient level, moisture content, sorbitol concentration, and ionic environment within a common hydrocolloid framework, rather than as direct effects of protein source alone.

The flow behavior of both formulations was successfully described by the Herschel–Bulkley model, confirming a yield-stress, shear-thinning response well suited to extrusion-based 3D food printing. The pea protein ink showed K=48.42±10.00 Pa·s^n^, $\sigma_{0}=119.56\pm 18.84$ Pa, and n=0.35±0.10, whereas the chickpea ink exhibited K=42.91±16.13 Pa·s^n^, $\sigma_{0}=158.10\pm 18.17$ Pa, and n=0.35±0.06. The similarly low flow index in both systems indicates pronounced shear thinning, implying a strong reduction in apparent viscosity as shear rate increases. This is desirable during printing because it facilitates stable flow through the nozzle at the high shear rates typical of extrusion, while the increase in viscosity at low shear supports filament formation after deposition [17,18].

The main difference between formulations was captured by the yield stress. The CPC-based ink displayed the higher *σ*_0_, indicating greater resistance to flow initiation and a more structured material at rest. Within the present design, this behavior is most plausibly related to its lower moisture content, higher ingredient loading, and formulation-specific ionic environment. From a printing standpoint, higher yield stress can contribute to reduced slumping and lateral spreading immediately after deposition. However, yield stress alone should not be taken as a direct predictor of overall printing performance, because post-shear rebuilding and thermal stabilization also contribute to shape retention.

**Figure 1:**
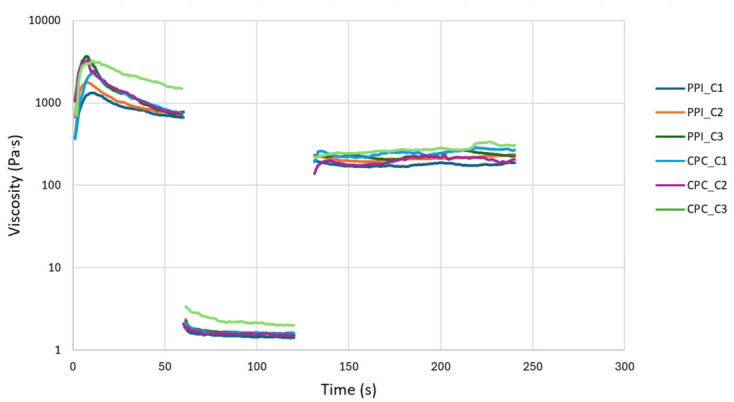
Thixotropy test for pea and chickpea-based food inks. PPI: Pea protein isolate. C1-C2-C3: cartridge number 1, 2 and 3. CPC: Chickpea protein concentrate. C1-C2-C3: cartridge number 1, 2 and 3.

Because extrusion printing is intrinsically time-dependent, combining rapid structural disruption in the nozzle with rebuilding after deposition, thixotropy was further evaluated using a three-interval thixotropy test (3ITT) (Figure 1).

Both inks exhibited high initial viscosities (10^3^–10^4^ Pa·s), consistent with structured soft-solid matrices at rest. Under high shear, viscosity dropped sharply to 1–10 Pa·s, indicating effective shear-induced fluidization and therefore favorable extrudability. Upon return to low shear, viscosity increased only partially, reaching approximately 200 Pa·s. Recovery ratios extracted from the curves were 19% for the pea protein ink and 16% for the chickpea ink, indicating limited structural rebuilding within the timescale of the test.

These recovery values should be interpreted critically. At 60 °C, both systems showed only modest isothermal recovery, which indicates that neither ink rapidly re-established its initial structure under the conditions of the 3ITT test. This represents a clear rheological limitation and suggests that self-support of the printed filament cannot be attributed to rapid structural rebuilding alone. At the same time, the 3ITT protocol was conducted at the syringe temperature and therefore does not fully reproduce the thermal transition occurring during actual printing.

In the printer, the inks were extruded from a syringe maintained at 60 °C into a cooler environment, and this temperature drop likely contributed to post-deposition stiffening. This point is particularly relevant in the presence of low-acyl gellan gum, whose structuring is strongly temperature-dependent [7,40].

Low-acyl gellan is fully solubilized at high temperature and, during cooling, undergoes gelation through the formation of double helices and their aggregation into ordered domains stabilized by ionic interactions. Under food-relevant ionic conditions, the sol–gel transition typically occurs within the range 45–65 °C, which is fully compatible with the thermal strategy adopted here [7,41,42].

Therefore, the low thixotropic recovery measured at 60 °C does not necessarily imply equally poor self-support after deposition. Rather, the ability of both inks to generate well-defined printed objects, likely resulted from the combined action of partial structural rebuilding, yield stress, and cooling-induced gel reinforcement. Since the printing bed temperature and cooling rate were not independently measured, this contribution should be regarded as a plausible interpretation rather than a directly demonstrated mechanism. It should also be noted that the 3ITT test itself could not be performed below 60 °C, because preliminary observations showed that gelation under those conditions prevented reliable testing.

The contrast between the two inks further indicates that yield stress at rest and reversibility after shear are distinct attributes. The chickpea ink combined the highest yield stress with the lowest recovery ratio, suggesting a structure that was more resistant to flow initiation but less capable of rebuilding after intense shear. This behavior is consistent with a denser, less hydrated, and possibly less reversible network. By contrast, the pea protein ink, despite its lower *σ*_0_, showed slightly greater recovery. In addition to its higher moisture content, this formulation contained a substantially higher sorbitol level, which may have acted as a plasticizer by increasing matrix mobility and reducing local brittleness within the protein–hydrocolloid network.

The observed differences should also be considered in light of protein–polysaccharide interactions. Both formulations contained the same hydrocolloid backbone, based on low-acyl gellan gum and xanthan gum, but differed in protein ingredient content, water level, sorbitol concentration, and salt origin. In such mixed systems, the final rheological response emerges from the combined effects of hydration, excluded-volume interactions, polymer compatibility, and ionic screening, rather than from protein concentration alone. At pH 7.2–7.5, both proteins and the anionic polysaccharides are predominantly negatively charged, making strong electrostatic attraction unlikely. Under these conditions, the system is more likely to be governed by water competition, segregative phase separation, and ion-mediated modulation of network formation [43,44].

**Figure 2:**
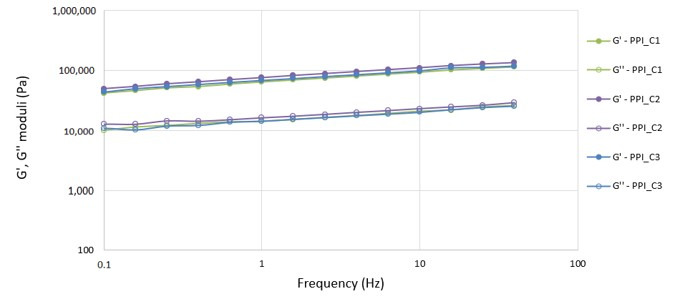
Mechanical spectra of pea-protein isolate based inks (PPIs). G′: Storage modulus. G″: Loss modulus, moduli are shown with filled and open markers, respectively; C1-C2-C3: cartridge number 1, 2, and 3.

**Figure 3:**
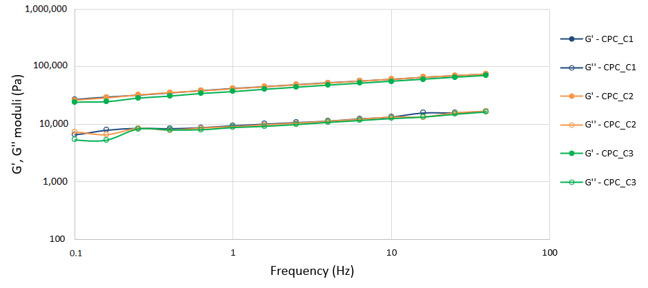
Mechanical spectra of chickpea-protein concentrate based inks (CPCs). G′: Storage modulus. G″: Loss modulus, moduli are shown with filled and open markers, respectively; C1-C2-C3: cartridge number 1, 2 and 3.

Oscillatory measurements were then used to further elucidate the structural organization of the inks under small deformations. Both formulations exhibited spectra characteristic of weak-gel systems, with G′ consistently higher than G″ across the investigated frequency range and no crossover point within the experimental window (Figure 2 and Figure 3). This elastic-dominant behavior confirms the presence of an interconnected network at rest and is consistent with the yield-stress behavior observed under steady shear.

The pea protein-based ink displayed slightly higher values of both G′ and G″, indicating a somewhat more cohesive viscoelastic structure under small-amplitude deformation. To interpret these spectra more rigorously, the complex modulus was fitted using the Bohlin weak-gel model, $G^{*}=A_{f}\omega^{1/z}$. The use of this model is justified by the typical weak-gel character of the mechanical spectra, namely elastic dominance, absence of crossover, and power-law frequency dependence, which are common features of food systems structured by weak physical interactions [29]. In this framework, *A_f_* describes gel strength, whereas *z* provides information on the extent of network coordination.

The Bohlin analysis showed a substantially higher gel strength parameter for the pea ink ($A_{f}=65.69\pm 5.59$ kPa) than for the chickpea ink ($A_{f}=38.97\pm 2.08$ kPa), whereas the interaction coefficient was very similar in the two systems (z=5.47±0.30 for the pea ink and z=5.23±0.08 for the chickpea ink). This indicates that both inks had a broadly similar spectral architecture but differed in the overall strength of the load-bearing network. These results help reconcile the apparent discrepancy between steady-shear and 3ITT data: the chickpea ink was more resistant to the onset of irreversible flow, whereas the pea ink developed a stronger and more cohesive weak-gel structure under oscillatory loading. For clarity, the main rheological parameters obtained from steady shear, thixotropy, and oscillatory measurements are summarized in Table 3.

The ionic environment likely contributed to these formulation-dependent differences. In both inks, pH values were well above the isoelectric points of the corresponding proteins. The pea protein-based ink showed a pH of 7.2, whereas the chickpea protein-based ink showed a pH of 7.5. Therefore, pH alone is unlikely to account for the observed rheological differences.

More relevant is the sodium environment. In the pea-based system, NaCl was added during preparation, whereas in the chickpea-based system the salt was already present in the protein ingredient. Even with comparable overall NaCl content, the two formulations may therefore have differed in effective ionic conditions because of differences in mineral profile, buffering capacity, and local ion distribution.

Moreover, since low-acyl gellan gelation is ion-mediated, these differences may have influenced not only yield stress, but also the reversibility of the structured network after shear and the final viscoelastic strength of the inks [45,46,47].

Overall, the rheological data indicate that both inks fell within a printable weak-gel window, but they achieved printability through different formulation routes. The CPC-based ink was characterized by higher resistance to flow onset and is therefore expected to better resist immediate collapse after extrusion. The PPI-ink, in contrast, exhibited slightly greater post-shear recovery and markedly higher weak-gel strength under oscillatory loading, suggesting improved filament cohesion and post-deposition consolidation.

### 3.3. 3D Printing of Soft-Solid Foods

The 3D printing of soft-solid foods was performed following a standard digital-to-physical workflow, in which a CAD model is converted into printer instructions (slicing and g-code generation) and subsequently fabricated by layer-by-layer extrusion. Because the dimensional accuracy and self-supporting ability of printed gels depend strongly on the interplay between ink rheology and process settings, printing parameters were selected to ensure continuous extrusion, interlayer adhesion, and acceptable shape retention of the deposited filaments [48,49,50,51]. More broadly, recent reviews on gel-based edible inks have emphasized that successful printing depends not only on flow behavior during extrusion, but also on the combined effects of gelation triggers, post-deposition stabilization, and process control [52,53].

Key controllable parameters included nozzle diameter, layer height, extrusion flow rate, nozzle speed, and infill geometry, all of which influence filament continuity, layer stacking, porosity, and dimensional stability [52].

Infill represents the internal filling structure of a 3D-printed object, defined by a geometric pattern and a specific density; by modulating material distribution and void arrangement, it can influence both mechanical and sensory properties of the final product [54,55,56,57]. In the present study, infill was kept constant at 20% and the same pattern was applied to all samples, so that both PPI- and CPC-based systems were compared under identical geometric conditions defined by the slicing software.

Temperature control was particularly critical because of the thermally triggered gelation of the inks: the syringe temperature was maintained at 60 °C to preserve a flowable state during extrusion while promoting rapid setting after deposition. In gellan-containing systems, the temperature drop after extrusion is known to contribute to curing and structural fixation, and this mechanism likely played a relevant role in the present formulations [53].

**Figure 4:**
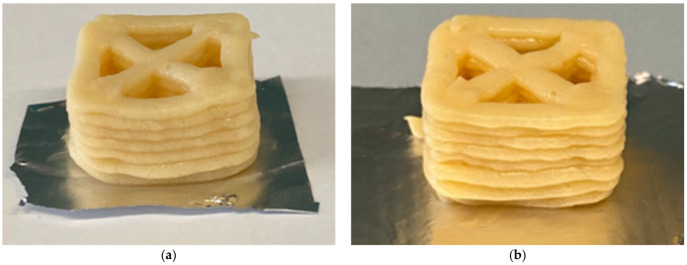
(**a**) = Pea protein-based printed object. (**b**) = Chickpea protein-based printed object.

A simple square geometry (20 × 20 × 10 mm) was selected to minimize geometric complexity and better isolate material effects. Under these selected conditions, both the pea protein- and chickpea-based inks enabled continuous and reproducible extrusion, producing self-supporting objects with regular external contours and visible multilayer stacking (Figure 4).

However, the printed objects did not show perfect geometrical fidelity. In particular, the internal infill pattern appeared less sharply defined than the nominal CAD design, and this loss of definition was more evident in the chickpea-based construct. This observation suggests that, although the selected conditions were suitable for continuous printing and gross shape retention, further improvement is still needed in terms of internal architecture resolution and dimensional precision. Thus, the present results should be interpreted as evidence of successful proof-of-concept printing rather than as a fully optimized manufacturing outcome.

Printed objects were then subjected to compression testing to quantify mechanical response relevant to handling and early oral processing.

Mechanical compression tests were performed at ambient temperature on printed specimens that had been stored overnight at 4 °C in sealed containers. This storage step was introduced to allow full structural stabilization before testing and to ensure reproducible comparison between samples. At the same time, overnight storage at a low temperature may have affected the final gel structure and, consequently, the measured mechanical properties. Cooling and holding may have promoted additional gellan structuring, water redistribution, and strengthening of interlayer contacts after deposition. Therefore, the compression results reported here should be interpreted as the response of stabilized printed objects rather than as the immediate post-printing mechanical state.

This distinction is relevant because post-processing and post-deposition evolution are increasingly recognized as important determinants of final shape fidelity and texture in gel-based printed foods [52,53].

**Figure 5:**
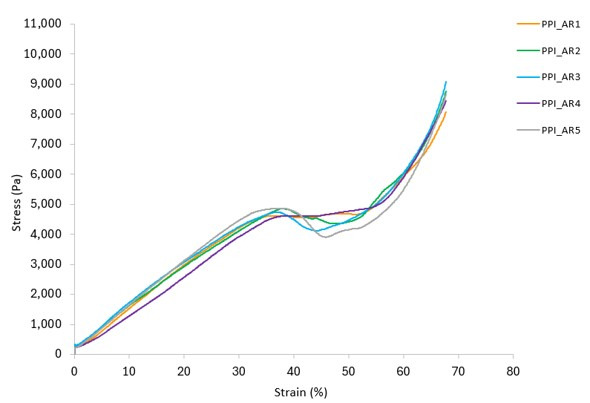
Compression test of pea protein isolate based (PPI) 3D-printed soft gels. AR1–AR5 represent the mean stress–strain curves of the first to fifth replicates, respectively.

**Figure 6:**
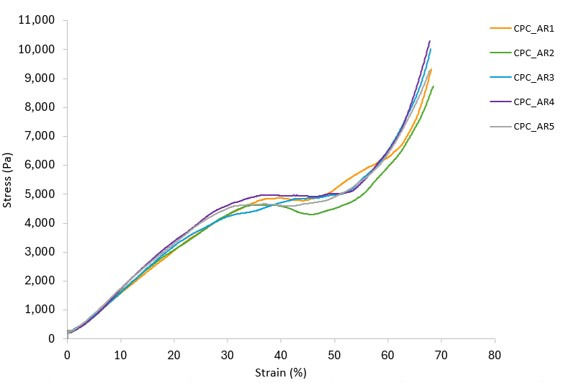
Compression test of chickpea protein concentrate based (CPC) 3D-printed soft gels. AR1–AR5 represent the mean stress–strain curves of the first to fifth replicates, respectively.

Samples were compressed at 1 mm/s up to 70% strain. As shown in Figure 5 and Figure 6, both the pea-based and chickpea-based printed objects displayed a characteristic three-stage compressive behavior: (i) an initial quasi-elastic region up to 30–35% strain, (ii) a plateau region between 35 and 55% strain, and (iii) a final yielding/densification phase at higher deformation. A consistent feature across replicates was a localized stress drop within the intermediate portion of the curve. This phenomenon is attributable to the internal architecture of the printed structures, which were manufactured with 20% infill rather than as fully dense solids. Under compression, partial collapse and reorganization of the porous, layered network can temporarily reduce load-bearing capacity, after which stress rises again as the structure compacts and progressively densifies [58,59].

To quantitatively compare the mechanical responses, three descriptors were extracted from the stress–strain curves: elastic modulus, compressive yield stress, and specific deformation energy. Results obtained from the samples described in Section 2.7 are reported in Table 4.

Both formulations exhibited similar stiffness, although the CPC-based gel showed a slightly higher modulus (139.84 ± 24.00 Pa) than the PPI-based gel (130.08 ± 12.54 Pa), suggesting a marginally more compact and rigid network under small deformation. In contrast, the PPI-based printed gel exhibited a higher compressive yield stress (4694.65 ± 900.45 Pa) than the CPC-based gel (4509.92 ± 887.71 Pa), indicating greater resistance to the onset of irreversible deformation. Energy-related properties further differentiated the two systems: the CPC-based gel showed a higher specific deformation energy (3472.97 ± 394.19 J/m^3^) than the PPI-based gel (3029.08 ± 223.44 J/m^3^), indicating a greater capacity to absorb mechanical energy before structural failure.

Overall, both printed gels exhibited mechanical profiles compatible with extrusion-based manufacturing of soft-solid foods. Within the tested conditions, the PPI formulation provided greater resistance to irreversible deformation, whereas the CPC formulation presented slightly higher stiffness and toughness, reflecting differences in network compaction and energy dissipation mechanisms. According to the statistical analysis performed, no significant differences were observed among replicates (*p* > 0.05), indicating good repeatability within each formulation (Table 4).

The compression results are broadly consistent with the rheological profile discussed in Section 3.2: the CPC ink exhibited a higher yield stress in steady shear, whereas the PPI ink showed a more cohesive viscoelastic network and slightly greater thixotropic recovery, which may reflect improved post-deposition consolidation and interlayer integrity after stabilization.

## 4. Conclusions

This study investigated the rheology, printability, and texture of plant protein–hydrocolloid soft gels for extrusion-based 3D printing using two realistic formulation systems based on commercial pea protein isolate and chickpea protein concentrate. Rather than isolating protein source as a single variable, the work compared two complete inks designed to achieve comparable nutritional positioning and printable behavior within the same hydrocolloid framework.

Both inks exhibited rheological characteristics compatible with extrusion-based printing, including yield stress, shear-thinning flow, and weak-gel viscoelasticity. However, the two systems achieved printability through different formulation routes. The CPC-based ink showed higher resistance to flow initiation, whereas the pea-based ink exhibited slightly greater post-shear structural recovery and higher weak-gel strength under oscillatory loading.

Under the selected printing conditions, both formulations enabled continuous extrusion and the fabrication of self-supporting soft-gel objects. At the same time, the limited definition of the internal infill pattern, particularly in the CPC-based constructs, indicates that further improvement in geometric fidelity is still required. Compression testing on stabilized printed samples showed that the CPC formulation produced slightly stiffer and tougher gels, whereas the pea formulation showed greater resistance to irreversible deformation.

Overall, the results indicate that printability and final texture in these systems were governed primarily by formulation architecture, including solids content, hydration state, hydrocolloid structuring, sorbitol level, and ionic environment. The hydrocolloid backbone defined the printable domain, whereas the differences between the two inks emerged from the overall formulation design. The present work therefore provides a materials-oriented basis for the development of plant-based printed soft foods, while also highlighting the need for further refinement of printing conditions and post-deposition stabilization analysis.

## Figures and Tables

**Table 1:** Main outcomes of the preliminary experimental trials carried out to optimize ink preparation and 3D printing conditions. For each parameter, the tested range, observed limitations, final selected condition, and selection criterion are reported.

Parameter	Tests Performed	Observed Issue	Final Selected Condition	Motivation
Ingredient selection	—	Non-optimized formulations produced non-printable inks (too fluid or too viscous)	—	—
Mixing temperature	50–70 °C	<60 °C caused premature gelation	60–62 °C	Compromise between fluidity and stability
LA Gellan dissolution	70–90 °C	<80 °C incomplete dissolution	80 °C	Complete solubilization
Stirring speed	100–1000 rpm	>220 rpm caused air incorporation	200 rpm	Minimal bubble formation
Printing speed	5–15 mm/s	>8 mm/s discontinuous filament	7 mm/s	Continuous extrusion
Flow rate	80–110%	90–100% over-extrusion	80%	Geometrical fidelity

**Table 2:** Pea and chickpea protein powder characterization: PPI: Pea protein isolate, CPC: chickpea protein concentrate, TS: total solids, WHC: water holding capacity, OHC: oil holding capacity, FC: foaming capacity, EC: emulsion capacity, ES: emulsion stability.

	Protein (%)	TS (%)	WHC (gH_2_O/gTS)	OHC (gOil/gTS)	FC (%)	EC (%)	ES (%)
PPI	78.75 ± 0.35	93.32 ± 0.33	1.76 ± 0.01	1.32 ± 0.11	7.74 ± 0.09	72.19 ± 1.33	60.31 ± 0.44
CPC	68.50 ± 0.70	94.14 ± 0.26	1.17 ± 0.11	1.41 ± 0.51	2.27 ± 0.56	68.84 ± 1.64	58.36 ± 3.20

**Table 3:** Summary of steady-shear, thixotropic, and oscillatory rheological parameters of PPI- and CPC-based inks.

Parameter	Unit	PPI Ink	CPC Ink
Consistency index, K	Pa·sn	48.42 ± 10.00	42.91 ± 16.13
Yield stress, σ0	Pa	119.56 ± 18.84	158.10 ± 18.17
Flow behavior index, n	–	0.35 ± 0.10	0.35 ± 0.06
Thixotropic recovery	%	19	16
Storage modulus, G′ at 1 Hz	kPa	67.73 ± 4.87	39.97 ± 2.66
Loss modulus, G″ at 1 Hz	kPa	14.42 ± 1.38	9.06 ± 0.32
tan δ at 1 Hz	–	0.21	0.23
Bohlin gel strength, Af	kPa	65.69 ± 5.59	38.97 ± 2.08
Bohlin interaction coefficient, z	–	5.47 ± 0.30	5.23 ± 0.08

**Table 4:** Mechanical properties obtained from the compression curves for pea- and chickpea-based formulations showed no statistically significant differences among samples, as evidenced by identical superscript letters across all groups (*p* > 0.05). EM: elastic modulus, YS: yield stress, SDE: specific deformation energy. AR1–AR5 represent the mean stress–strain curves of the first to fifth replicates, respectively.

	EM (Pa)	YS (Pa)	SDE (J/m^3^)		EM (Pa)	YS (Pa)	SDE (J/m^3^)
PEA_AR1	134.89 ± 13.27 ^a^	4679.49 ± 303.72 ^a^	3013.98 ± 236.77 ^a^	CP_AR1	128.48 ± 33.89 ^a^	4525.94 ± 1338.57 ^a^	3142.19 ± 453.86 ^a^
PEA_AR2	124.64 ± 1.33 ^a^	4973.65 ± 285.57 ^a^	3042.90 ± 56.87 ^a^	CP_AR2	132.79 ± 20.03 ^a^	4415.51 ± 605.22 ^a^	2894.16 ± 424.98 ^a^
PEA_AR3	128.25 ± 7.89 ^a^	4812.33 ± 166.00 ^a^	3074.83 ± 65.69 ^a^	CP_AR3	141.83 ± 13.92 ^a^	4000.70 ± 621.38 ^a^	3145.03 ± 363.01 ^a^
PEA_AR4	125.02 ± 10.27 ^a^	4067.72 ± 1791.41 ^a^	2977.59 ± 213.68 ^a^	CP_AR4	153.01 ± 34.30 ^a^	4776.28 ± 1184.86 ^a^	3369.27 ± 440.47 ^a^
PEA_AR5	137.61 ± 20.01 ^a^	4940.04 ± 798.76 ^a^	3036.09 ± 417.19 ^a^	CP_AR5	145.27 ± 10.68 ^a^	4831.17 ± 302.34 ^a^	3314.21 ± 152.64 ^a^
Pea AR1-AR5 Mean	130.08 ± 12.54	4694.65 ± 900.45	3029.08 ± 223.44	CP AR1-AR5 Mean	139.84 ± 24.00	4509.92 ± 887.71	3472.97 ± 394.19

## Data Availability

The data that support the findings of this study are available from the corresponding author upon reasonable request.
